# Critical roles for Akt kinase in controlling HIV envelope-mediated depletion of CD4 T cells

**DOI:** 10.1186/1742-4690-10-60

**Published:** 2013-06-06

**Authors:** Haishan Li, C David Pauza

**Affiliations:** 1 Institute of Human Virology and Department of Medicine, University of Maryland School of Medicine, Baltimore, MD 21201, USA

**Keywords:** HIV, Envelope, Akt, p38, CD4 T cell death, CCR5, CD4, Antiviral therapy

## Abstract

**Background:**

The cell surface receptors CD4 and CCR5 bind CCR5-tropic HIV Envelope (Env) glycoprotein during virus attachment. These same receptors have signaling activities related to normal immune cell functions. We also know that Env binds to CCR5 present at high levels on CD4-negative γδ T cells where it signals through p38 MAP kinase to activate caspases and Fas-independent cell death. Here, we asked whether Env signaling through cellular receptors is responsible for death among uninfected CD4+/CCR5+ T cells and what are the effects of Env on CD4+/CCR5-negative cells that might impact HIV infection. The outcomes of Env binding are analyzed in terms of signal transduction and the effects on cell activation or cell death pathways.

**Results:**

Env binding to CD4 signals through Erk and Akt kinases. Activation of Erk/Akt suppresses p38 due to CCR5 binding, and allows cell survival. When CD4 signaling was blocked by soluble CD4 or protein kinase inhibitors, p38 activation and Fas-independent cell death were increased among uninfected CD4+ CCR5+ T cells. We also noted specific effects of CD4 signaling on CCR5-negative CD4 T cells in tonsil lymphocyte cultures. Exposure to CCR5-tropic HIV Env (BaL strain) increased expression of CXCR5, PD-1, Fas and FasL. Among CD4+/CCR5- T cells expressing high levels of CXCR5 and PD-1, there were substantial amounts of Fas-dependent cell death. Increased CXCR5 and PD-1 expression was blocked by soluble CD4 or specific inhibitors of the Akt kinase, showing a direct relationship between CD4 signaling, T cell activation and Fas-dependent cell death.

**Conclusions:**

Specific inhibition of Akt activation increased Env-dependent cell death of CCR5+ CD4 T cells. The same inhibitor, antibodies blocking the CD4 binding site on gp120, or soluble CD4 also prevented the increase in expression of CXCR5 or PD-1, and reduced the levels of Fas-dependent cell death. The Akt kinase and related signaling events, are key to cell survival that is needed for productive infection, and may be targets for the development of antivirals. Specific inhibitors of Akt would decrease productive infection, by favoring cell death during virus attachment to CD4+ CCR5+ target cells, and reduce immune activation to prevent Fas-dependent death of uninfected CXCR5+ PD-1+ CD4 T cells including T follicular helper cells that share this phenotype.

## Background

HIV disease is characterized by CD4 T cell depletion and progressing immunodeficiency [1]. Because HIV infects only a small proportion of CD4 T cells (estimated at 0.1 ~ 1%) [2-4], much of the observed cell death is due to indirect or bystander effects [4,5]. In fact, the majority of T cells undergoing apoptosis in peripheral blood, lymph nodes, thymus or spleen from HIV-infected patients or SIV-infected macaques were not infected [6-9]. Several mechanisms have been proposed for uninfected, bystander CD4 T cell depletion, including direct action of HIV proteins, activation-induced cell death, autologous cell-mediated cytotoxicity against uninfected T cells, and dysregulation of cytokine/chemokine production [4,10,11]. Several of these mechanisms implicate HIV envelope (Env) glycoprotein as a promoter of uninfected CD4 T cell depletion [12].

We wanted to understand the effects of CCR5-tropic HIV Env signal transduction through CD4 or CCR5. Normally, these signaling receptors are involved in controlling immune responses. Env binding will also trigger signal transduction and may affect HIV infection and virus replication. In fact, when R5-tropic Env glycoprotein binds CCR5 on CD4-negative γδ T cells, p38 MAP kinase is activated, caspase activity increased and Fas-independent cell death resulted [13,14]. It was also reported that HIV Env glycoprotein (from HIV-1 strains IIIB, Bal, MN, JRFL, SF2 and SF162) induced apoptosis of uninfected, CD4-negative neurons (strains IIIB, SF2 and SF162) [15], cardiomyocytes (strain JR-FL) [16], hepatocytes (strain MN) [17], proximal renal tubular cells [18], lung endothelial cells (strains BaL and MN) [19] and human vascular endothelial cells [20].

The mechanisms for Env-induced cell death are controversial [12,21,22]. Early studies proposed that oligomeric or particle-associated Env cross-links CD4 which increases spontaneous cell apoptosis, activation-induced cell death and cell susceptibility to Fas-dependent apoptosis [12]. Others argued against a direct role for CD4 in the pathway for cell death. It was reported that Env induced apoptosis only in T cell lines lacking a CD4 cytoplasmic domain [23] and Env mutants that bind CXCR4 but do not bind CD4, still induced apoptosis compared to mutants defective for CXCR4 binding that did not cause cell death [24]. Env-dependent CD4 T cell death was blocked by CCR5 or CXCR4 binding antagonists [25-27] and soluble CD4 (sCD4) increased R5 or X4-induced CD4 T cell death [21,22].

Our studies focused on signal transduction events driven by HIV Env binding to cell surface receptors on tonsil CD4 T cells. We are defining discrete signaling events after CD4 or CCR5 binding, and studying cross-regulation among these pathways to learn more about the function of each major HIV receptor beyond their established roles in virus penetration. Receptor signaling may be involved in both indirect cell death and the control of productive infection. By targeting protein kinases involved in signal transduction, using small-molecule inhibitory drugs already in clinical development for cancer therapy, we may identify new targets for antiretroviral agents among host cell pathways.

## Results

### HIV R5-tropic Env induces tonsil CD4 T cell death

**Figure 1:**
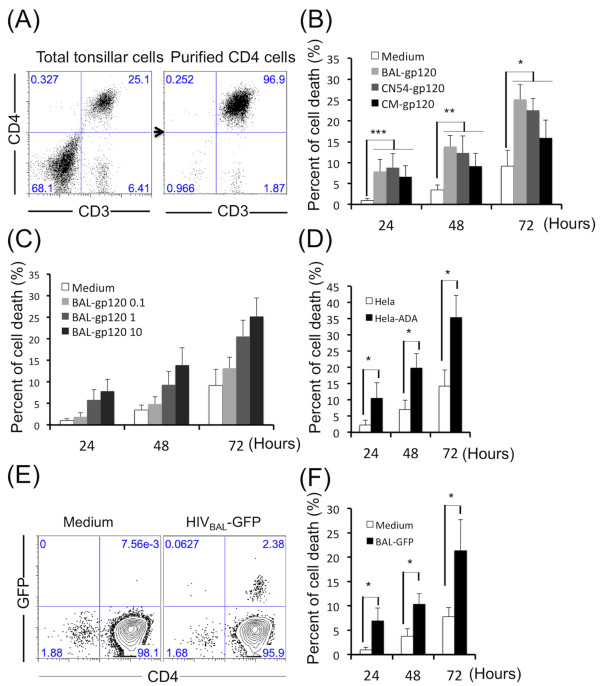
**HIV R5-tropic Env induces tonsil CD4 T cell death. **(**A**) Tonsil CD4 T cells were purified by negative selection. (**B**) Purified tonsil CD4 T cells were treated with or without soluble gp120 (BaL, CN54 or CM) at 10 μg/ml (n = 7); or (**C**) treated with BaL-gp120 at different concentrations (μg/ml); or (**D**) were cocultured with HeLa cells or HeLa cells expressing HIV ADA-envelope (HeLa-ADA) at a ratio of 1:2 (n = 4); or (**F**) treated with pseudo virion expressing HIV BaL envelope and GFP at 50 ng/ml of p24 (n = 4) for 3 days. Cell death was evaluated every 24 hours. The statistical significance was analyzed with Student’s t test; *P < 0.05, **P < 0.005, ***P < 0.0001. (**E**) Purified tonsil CD4 T cells were treated with pseudo-viruses expressing HIV BaL envelope and GFP at 50 ng/ml of p24 for 24 hours. The GFP positive cells were detected by flow cytometry. Data are representative of four independent experiments.

We first tested whether HIV R5 tropic Env kills human tonsil CD4 T cells. Fresh, CD4 T cells were purified by negative selection from dissected human tonsils (Figure 1A). We then used soluble gp120, cell-associated Env or virions to test the killing effect of HIV Env. For soluble gp120, purified CD4 T cells were treated with one of three soluble R5-tropic HIV gp120 proteins: BaL (clade B), CN54 (B’/C recombinant) or CM (clade E) at 10 μg/ml for 3 days. Cell death was evaluated every 24 hours. Each of the soluble gp120 proteins showed significant killing of CD4 T cells (Figure 1B). By 24 hours, 5-10% of CD4 T cells were killed which was significant compared to controls. Longer incubation times caused greater cell death. By 72 hours, we observed 20-30% of CD4 T cells were dead. Significant cell killing was also observed with soluble Env at 1 or 10 μg/ml. The effect was reduced at Env concentrations below 1 μg/ml (Figure 1C).

We next examined the effects of cell- or virion-associated HIV Env. A stable HeLa cell line expressing HIV envelope from the ADA strain (HeLa-ADA) provided cell-associated Env. Cell lines HeLa or HeLa-ADA were mixed in a ratio of 1:2 with purified tonsil CD4 T cells. HeLa-ADA induced significant CD4 T cell death compared with HeLa cell control at both early (24h) and late times (72h) (Figure 1D). A pseudovirus expressing HIV BaL Env and GFP was used to evaluate the effect of virion-associated Env. Although only 2.4% of CD4 cells became infected (Figure 1E), virion preparations induced on average, 22% cell death within 72 hours (Figure 1F). The fusion inhibitor T20 did not prevent cell killing by either cell- or virion-associated Env (data not shown).

### Distinct roles for CD4 and CCR5 in Env-induced CD4 T cell death

**Figure 2:**
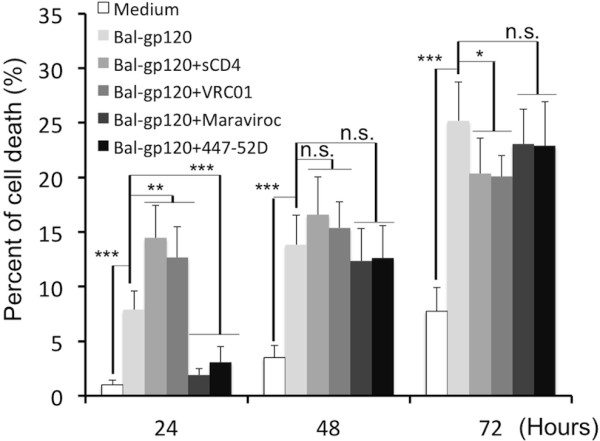
**CD4 and CCR5 have different effects on HIV Env-induced CD4 T cell death. **Cell death was evaluated every 24 hours after purified tonsil CD4 T cells were incubated with soluble HIV gp120 protein (BaL, 10 μg/ml) in the absence or presence of blocking reagents for CD4 (sCD4 or VRC01, 20 μg/ml) or CCR5 (Maraviroc 1 μM; or 447-52D Ab 20 μg/ml) binding for 3 days (n = 7). The statistical significance was analyzed. *P < 0.05, **P < 0.005, ***P < 0.0001 (Student’s t test).

We hypothesized that cell death induced by Env depended on CD4 or CCR5-mediated signaling. To test this idea, we blocked Env binding to CD4 with soluble CD4 (sCD4) or neutralizing antibody VRC01 which targets the CD4 binding site on Env. Env-CCR5 binding was blocked by the CCR5 antagonist Maraviroc or neutralizing antibody 447-52D that blocks the co-receptor binding site on Env. When Env-CD4 interactions were blocked, cell death increased significantly (Figure 2). Adding Maraviroc or antibody 447-52D at the start of culture, reduced cell depletion at the 24 hour interval (Figure 2).

### CD4 and CCR5 mediated different signaling

We wanted to understand why blocking Env binding to CCR5 inhibited but blocking Env-CD4 interactions actually increased cell death. We hypothesized that Env-CD4 binding induced survival signals that counteracted or directly inhibited the death signal generated by Env binding to CCR5.

**Figure 3:**
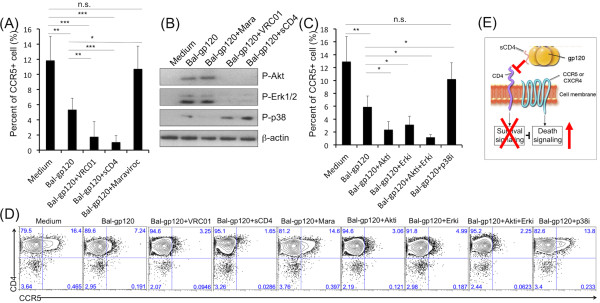
**CD4 and CCR5 mediated different signaling. **(**A**) Purified tonsil CD4 T cells were incubated with soluble HIV gp120 protein (BaL, 10 μg/ml) in the absence or presence of VRC01 (20 μg/ml), sCD4 (20 μg/ml) or Maraviroc (1 μM) for 24 hours (n = 4). Frequency of CCR5+ cells was measured by flow cytometry. The statistical significance was analyzed. *P < 0.05, **P < 0.005, ***P < 0.0001 (Student’s t test). (**B**) Purified tonsil CD4 T cells were incubated with or without BaL-gp120 for 1 h on ice. Thereafter, cells were incubated at 37°C for 2 minutes to induce stimulation. (**C**) Purified tonsil CD4 T cells were untreated or pretreated with specific inhibitors (2μM) for Akt (LY294002), Erk (U0126) or p38 (SB203580) for 1 hour at 37°C before incubated with BaL gp120 (10 μg/ml) for 24 hours (n = 4). Frequency of CCR5+ cells was measured by flow cytometry. The statistical significance was analyzed. *P < 0.05, **P < 0.005, ***P < 0.0001 (Student’s t test). (**D**) A representative result of CCR5+ cells in tonsil CD4 T cells in different culture conditions as described above for 24 hours. (**E**) A proposed model based on our findings that Env-CD4 binding induced survival signals (Akt and Erk) that counteracted or directly inhibited the death signal (p38) generated upon Env binding to CCR5.

To test this hypothesis, we examined CCR5+ cell depletion at 24 h. In our study, individual donors had 7-17% of tonsil CD4 T cells that also expressed CCR5+ (Figure 3A, D). The BaL-gp120 depleted on average, 55% of the CCR5+ CD4 T cells within 24 hours (Figure 3A, D). Adding soluble CD4 or VRC01 monoclonal antibody increased the rate of CCR5+ cell loss, while Maraviroc blocked cell depletion (Figure 3A, D). We next examined signaling pathways activated when Env binds to CD4 or CCR5. We confirmed that BaL-gp120 activated Akt, Erk and p38 signaling in tonsil CD4 T cells (Figure 3B). Soluble CD4 or VRC01 antibody inhibited Akt or Erk activation, but enhanced phosphorylation of p38 (Figure 3B). Maraviroc inhibited Env-dependent p38 activation, but did not affect Akt or Erk (Figure 3B).

Next, we used specific signal transduction inhibitors to test the roles for individual pathways in Env-mediated killing of CD4 T cells. All inhibitors were used at concentrations which had no measurable cytotoxicity. Adding Akt (LY294002) or Erk (U0126) inhibitors increased Env-dependent CCR5+ cell depletion (Figure 3C, D). When Akt and Erk inhibitors were combined, nearly all CCR5+ cells were depleted after Env exposure (Figure 3C, D). A p38 inhibitor (SB203580) reduced CCR5+ cell depletion (Figure 3C, D). These results support a mechanism for HIV Env-mediated killing of uninfected CD4 T cells that depends on Env signaling through CCR5, but that signal can be modulated when Env binds CD4 and limits the extent of cell death (Figure 3E).

### A subset of CCR5-negative CD4 T cells in tonsil express activation markers and are susceptible to Fas-mediated killing

**Figure 4:**
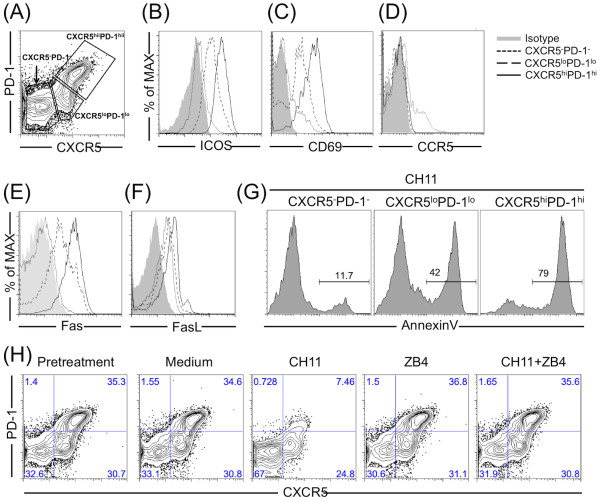
**Germinal center CXCR5 + PD-1+ CD4 T cells are susceptible to Fas-mediated cell death. **(**A**) Tonsil CD4 T cell subsets were characterized on the basis of CXCR5 and PD-1 expression. (**B**-**F**) Expression levels of ICOS, CD69, CCR5, Fas or FasL on subsets were examined by flow cytometry. (**G**, **H**) Purified tonsil CD4 T cells were treated with or without active anti-Fas antibody CH11 (1 μg/ml), or neutralizing anti-Fas antibody ZB4 (1 μg/ml), or both for 8 hours (**G**) or 24 hours (**H**). Cell apoptosis was determined by expression of Annexin V (**G**) and the frequency of different subsets was measured by expression of CXCR5 and PD-1 (**H**). Data are representative of three independent experiments.

**Figure 5:**
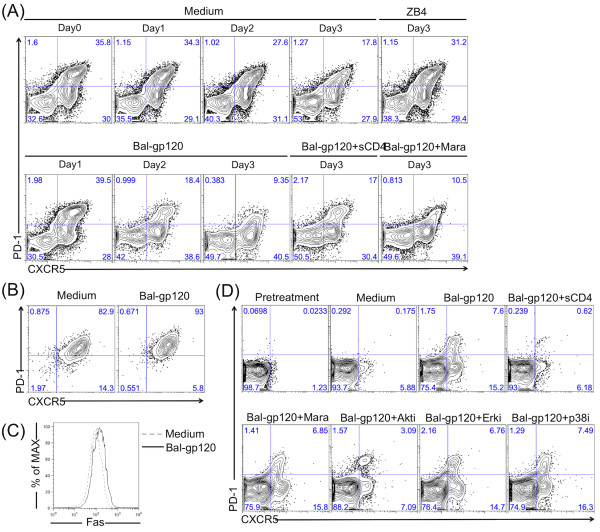
**Env-dependent CD4-mediated activation of Akt increases CXCR5/PD-1 expression and Fas-dependent cell death. **(**A**) Purified tonsil CD4 T cells were treated with or without BaL-gp120 for 3 days. In some cases, neutralizing anti-Fas antibody ZB4 (1 μg/ml), sCD4 (10 μg/ml) or Maraviroc (1 μM) was added to the T cell cultures. Expression of CXCR5 and PD-1 were monitored every day by flow cytometry. (**B**, **C**) CXCR5^hi^PD-1^hi ^cells were purified by cell sorting and cultured in the absence or presence of BaL-gp120 for 3 days. Expression of CXCR5, PD-1 (**B**), or Fas (**C**) was detected by flow cytometry. (**D**) CXCR5^-^PD-1^- ^cells were purified by cell sorting and cultured in the absence or presence of BaL-gp120 for 3 days. In some cases, sCD4 (10 μg/ml), Maraviroc (1 μM), or specific inhibitors (2 μM) for Akt, Erk or p38, were added to cell cultures. Expression of CXCR5 and PD-1 were detected by flow cytometry. Data are representative of three independent experiments.

Aside from the susceptible CD4+ CCR5+ T cells, 30-60% of tonsil CD4 cells express activation markers including CXCR5, PD-1 (Figure 4A), ICOS (Figure 4B) and CD69 (Figure 4C). These activated subsets do not express CCR5 (Figure 4D) [28] and thus resist Env-CCR5-mediated killing. However, they do express high levels of Fas (Figure 4E) and FasL (Figure 4F). Fas agonist antibody (CH11) induced high levels of apoptosis (Figure 4G) and the effect was blocked by Fas neutralizing antibody ZB4 (Figure 4H). The frequency of highly activated T cells (CXCR5^hi^PD-1^hi^) gradually declined during culture (from 36% to 18% in this typical example, Figure 5A); by 3 days, ~50% of the activated T cells were lost; Fas neutralizing antibody ZB4 inhibited this cell loss (Figure 5A). We did not observe significant changes in the frequency of less activated T cells (CXCR5^lo^PD-1^lo^) during this time course or after treatments (Figure 5A).

### HIV Env promotes activation and cell death among CCR5-negative cells

Having found that highly activated CXCR5^hi^PD-1^hi^ cells are a major subset of tonsil CD4 T cells and are susceptible to FasL-Fas-mediated apoptosis, we next wanted to define the effects of HIV Env. Purified tonsil CD4 T cells were incubated with or without BaL-gp120 for 3 days; CXCR5 and PD-1 expression were monitored daily by flow cytometry. Compared with controls, adding Env glycoprotein increased the proportion of activated cells by day 1, followed by a rapid decline in this subset on days 2 and 3 (Figure 5A). BaL-gp120 also increased the frequency of a less-activated subset at days 2 and 3 (Figure 5A). Soluble CD4 but not Maraviroc, prevented Env activation of T cells, pointing to Env-CD4 interactions as the major signaling mechanism. Since we used a CCR5-tropic Env (BaL) we did not expect it to bind CXCR4 on these activated T cells.

Because the activated cell subset (CXCR5^hi^PD-1^hi^) did not express CCR5 [28], early (proportional) increases in these cells might be due to the loss of CCR5+ T cells. To test the direct effect of Env on activated cells, we purified them and treated with BaL-gp120 for 3 days which enhanced expression of both CXCR5 and PD-1 (Figure 5B) and slightly elevated Fas expression (Figure 5C). This result shows that the increase in CXCR5^lo^PD-1^lo^ cells in response to Env signaling was not due to phenotypic reversion of highly active cells but was due to depletion of the activated subset.

We asked whether Env:CD4-mediated Akt or Erk signaling was required for CD4 T cell activation and especially for CXCR5 and PD-1 expression. We purified non-activated (CXCR5^-^ and PD-1^-^) cells and cultured them with BaL-gp120. After 3 days, both CXCR5^lo^PD-1^lo^ (15% of the total CD4 cells) and CXCR5^hi^PD-1^hi^ T cells (7%) (Figure 5D) were generated in these cultures; these cells also expressed higher Fas (data not shown). Soluble CD4 but not Maraviroc prevented Env-induced cell activation (Figure 5D). An inhibitor of Akt but not Erk phosphorylation, or a p38 inhibitor specifically blocked this pathway (Figure 5D).

HIV Env binds and signals through CD4; the signal leads to Akt phosphorylation and T cell activation with higher expression of CXCR5 and PD-1. Along with higher expression of Fas and FasL, these cells become more prone to apoptosis. This mechanism links HIV Env signaling with tonsil CD4 T cell death in CCR5-negative subsets.

## Discussion

We investigated the mechanisms for R5-tropic HIV Env-induced killing of tonsil CD4 T cells. Env binding to CCR5 activated p38 kinase and caspase resulting in death of CCR5+ cells during the first 24 hours of culture. However, Env binding to CD4 triggered Akt/Erk which modulated p38 activation and counteracted the death signal. The outcome of Env binding to CCR5+ cells was a balance of survival (Akt, Erk) versus death (p38) signaling. A distinct mechanism targeted CCR5-negative cells and required CD4 signaling through Akt pathways to promote T cell activation and cell killing by Fas-dependent apoptosis. Thus, Env:CD4 interactions (with CCR5-tropic Env glycoprotein) have different effects on CD4 cell subsets; first mitigating the impact of CCR5 signaling to reduce rapid, Fas-independent cell death and later promoting activation of CCR5-negative T cells leading to Fas-dependent cell death.

Previous studies showing that sCD4 enhanced HIV Env-induced CD4 T cell death [21,22], explained that sCD4 induced gp120 conformational changes that were required for chemokine receptor binding. At 1 μg/ml, gp120 is present at approximately 10-fold molar excess above cell surface CD4 receptors, and 100-fold molar excess above cell surface CCR5. gp120 binding to cell surface CD4 gains an advantage through avidity, for binding CCR5. If this advantage is only 10-fold, a very conservative estimate, gp120 bound to CD4 will out-compete solution gp120 for CCR5 binding, whether solution gp120 is bound to sCD4 or not. Consequently, gp120 bound to cell surface CD4 likely has a significant advantage over soluble CD4 for binding to CCR5, irrespective of the dissociation constants and even when sCD4 is in great excess.

Our study indicated that signaling through CD4, with activation of Akt/Erk kinase, actually modulates killing of CCR5+ cells. When Akt/Erk signaling is blocked by sCD4, VRC01 or specific inhibitors, the p38 death signal became stronger and cell death are more prominent because the tempering effect of signaling through cell surface CD4 is not present. During HIV attachment, gp120 binds CD4 and then CCR5 to initiate the entry pathway. In our view, viral evolution to CD4 binding may be a mechanism for modulating cell death due to chemokine receptor engagement; this adaptation increased the efficiency of infection and overall viral virulence. A mechanism that protects CD4+/CCR5+ T cells from indirect depletion, preserves this CD4 subset and positively selects for highly transmissible, CCR5-tropic HIV.

Another consequence of Env:CD4 signaling is T cell activation leading to Fas-mediated cell killing among CCR5-negative cells that escaped direct, p38-mediated killing. Where and when this occurs in vivo are key questions. During viremic HIV infection, it was estimated that soluble gp120 levels in blood ranged between 120–960 ng/ml and total gp120, including soluble, virion and cell-associated forms, is between 500 ng/ml and 5 ug/ml [29-33]. We expect local concentrations of gp120 in lymph nodes to be higher, especially around germinal centers where antigen normally accumulates. Virological synapses form during the attachment process, leading to co-localization of Env, CD4 and coreceptors that will further exaggerate signaling mechanisms beyond what we observe with soluble gp120. Consequently, lymphoid tissue CD4 T cells likely encounter soluble or virion-associated Env at concentrations sufficient to promote death of uninfected bystander cells. We showed that Env treatment increased CXCR5 and PD-1 expression in tonsil CD4 T cells, which are markers of T cell activation and also important for defining the T follicular helper cell (TFH) subset that is crucial for B cell differentiation and antibody production. Env glycoprotein, accumulating to high levels around germinal centers, would signal through CD4 and promote activation of TFH cells. Indeed, recent papers linked the accumulation of lymph node TFH with HIV disease. Among HIV-infected patients, viremia was associated with a shift in the distribution of lymph node CD4 cells with substantially elevated TFH subsets [34]. Increased TFH were coincident with hypergammaglobulinemia, a recognized consequence of HIV infection [35,36]. In SIV-infected macaques, lymph node TFH were elevated and also linked to higher antibody production [37]. If Env glycoprotein signaling contributes to the increased levels of TFH in lymph nodes, it might also promote CD4 cell depletion by increasing susceptibility to Fas:FasL induced cell death. This mechanism can be targeted with specific inhibitors of Akt activation.

Our system used purified tonsil CD4 T cells and CCR5-tropic HIV Env. Others tested a model for abortive infection of CXCR4+ tonsil CD4 cells where cytoplasmic viral DNA triggered a cell death pathway [38]. To avoid abortive infection, in our experiments, we used soluble gp120 and purified CD4 T cells; this allowed us to observe the unusual effects of Env-dependent Akt activation, and how we might exploit these pathways in new therapies. However, it will be important to learn whether identifiable CD4 T cell subsets may vary in their susceptibility to individual cell death pathways.

## Conclusions

We identified roles for Akt, Erk and p38 kinases in death of uninfected CD4+ T cells in vitro. Specific binding, signal transduction and protein kinase inhibitors were used to block pathologic effects of Env glycoprotein. Our studies emphasize the importance of focusing on Akt and Erk inhibitors to block CD4-dependent survival signaling and render cells more susceptible to CCR5-dependent cell killing. These same inhibitors prevented T cell activation that might be related to TFH over-production in lymph nodes during HIV infection. Inhibitors of Akt and Erk are already being used in therapies for cancer and autoimmune diseases; they may have value for treating HIV disease.

## Methods

### Tonsil cell isolation and tumor cell lines

Studies described here were approved by the Institutional Review Board at the University of Maryland, Baltimore. Tonsil samples were obtained from patients undergoing tonsillectomy. Single cells were collected after mechanical disruption of dissected tonsil and purification of mononuclear cells on density gradients (Ficoll-Paque; Amersham Biosciences). CD4 T cells were isolated by negative selection (Miltenyi Biotech). On average, 15% of total CD4 T cells from tonsil also expressed CCR5. Purified tonsil cells were cultured in RPMI 1640 supplemented with 10% fetal bovine serum (FBS; GIBCO), 2 mMol/L L-glutamine, and penicillin–streptomycin (100 U/ml and 100 mg/mL, respectively). HeLa cell lines were cultured in DMEM supplemented with 10% fetal bovine serum (FBS; GIBCO), 2 mMol/L L-glutamine, and penicillin–streptomycin (100 U/ml and 100 mg/mL, respectively). For HeLa-ADA cells expressing an R5 tropic HIV envelope (ADA), methotrexate was added to a final concentration of 2 μM.

### Preparation of pseudovirus and virus stocks

Pseudoviruses were prepared by co-transfecting 293 cells with an HIV BaL Env expression plasmid and HIV backbone plasmid expressing the entire HIV genome except Env (pNL4-3Δenv) with the aid of Lipofectamine™ 2000 (Invitrogen) according to the manufacturer's instructions. Pseudovirus stocks were harvested 48 hours after transfection, filtered (0.45-μm pore size), concentrated and stored at -80°C until used.

### Reagents

The following reagents were obtained through the AIDS Research and Reagent Program, Division of AIDS, NIAID, NIH: HIV CN54 gp120 from Dr. Ian Jones, HIV CM gp120 (Cat #2968), HIV gp120 MAb 447-52D, HIV gp120 MAb VRC01 from Dr. John Mascola, and the CCR5 binding antagonist drug Maraviroc (Cat # 11580). Other monoclonal antibodies include: activating Fas Ab (clone CH11, Millipore, Billerica, MA USA), blocking Fas Ab (clone ZB4, Millipore, Billerica, MA USA). PI3K/Akt inhibitor LY294002, Erk1/2 inhibitor U0126 and P38 inhibitor SB203580 were from Cell Signaling Technology, Inc., Danvers, MA.

### Cell death assays

The method was described previously [13]. Briefly, HeLa or HeLa-ADA cells were resuspended and mixed with purified CD4 T cells at 10^5^ HeLa cells: 2 × 10^5^ CD4 T cells, then plated in triplicate on 96-well plates for 3 days. The BaL (kindly provided by Dr. Tim Fouts, Profectus Biosciences, Baltimore, MD), CN54 or CM (NIH AIDS Research and Reference Program) gp120 proteins were added to 2 × 10^5^ CD4 T cells at varying concentrations in 96-well plates. After 3 days of culture, assays for cell death were done in triplicate and replicated with multiple, unrelated donors. For blocking studies against CD4 or CCR5, CD4 T cells were incubated with blocking reagents or antibodies for 1 hour at 37°C before adding to the killing assay. For inhibition studies with anti-gp120 antibodies, gp120 were pretreated with specific antibodies for 30 minutes at room temperature, then the mixtures were added to target cells for cytotoxicity assays. Signaling involved in cell killing was defined with specific inhibitors; CD4 T cells were incubated with signal transduction inhibitors for 1 hour at 37°C before the killing assay. The percentage of cell mortality was calculated according to the number of viable cells able to exclude trypan blue dye as follows: 1 – (total number of cells viable on day 2/total number of cells viable on day 1 immediately after stimulation) × 100. Cell death was confirmed with a cell death detection ELISA kit (Roche Diagnostics) or flow cytometry–based methods with AnnexinV and 7AAD staining.

### Immunoblot analysis

CD4 T cells were incubated with or without BaL-gp120 for 1 hour on ice. Thereafter, cells were incubated at 37°C for 2 minutes to induce stimulation. For blocking assays, cells were pretreated with Maraviroc for 30 minutes, or BaL-gp120 were pretreated with sCD4 for 30 minutes. Cell lysates were boiled for 10 minutes; proteins were separated by SDS-PAGE, transferred to nitrocellulose membranes and probed with primary antibodies. Secondary antibodies including HRP-conjugated, anti-rabbit, anti-rat, or anti-mouse (Cell Signaling Technology, Inc.), were visualized with enhanced chemiluminescence (GE Healthcare, Buckinghamshire, UK).

### Flow cytometry

Unless noted, cells were stained with fluorophore-conjugated monoclonal antibodies from BioLegend, San Diego, CA. Generally, cells were washed and resuspended in 50–100 μL of RPMI 1640, then stained with mouse anti-human CD4 (PE or FITC) clone OKT4, mouse anti-human CD3 (APC) clone UCHT1, Mouse anti-human PD-1 (FITC or APC) clone Eh12.2h7, mouse anti human CXCR5 (PerCP, BD Bioscience) clone Tg2/cxcr5, mouse anti-human ICOS (FITC) clone C398.4A, mouse anti-human CD69 (APC, BD Bioscience) clone Fn50, mouse anti-human CCR5-(APC, BD Bioscience) clone 2d7, mouse anti-human Fas (PE, BD Bioscience) clone Dx2, mouse anti-human FasL (PE) Nok-1 and isotype controls, including mouse IgG1-FITC clone X40, IgG1-PE clone X40, IgG1-PerCP clone X40, IgG1-APC clone X40, and Mouse IgG2b PerCP clone Mpc-11. Cells were stained to detect AnnexinV (PE) or 7AAD in Annexin V binding buffer. Data for at least 1 × 10^4^ lymphocytes (gated on the basis of forward- and side-scatter profiles) were acquired from each sample on a FACSCalibur flow cytometer (BD Biosciences). All samples were analyzed using FlowJo software (FlowJo 8.8.2, Tree Star, San Carlos, CA).

### Statistical analysis

Differences among groups were analyzed by Student’s t test. P < 0.05 was considered to be significant.

## Abbreviations

HIV-1: Human immunodeficiency virus type 1; TFH: T follicular helper cells.

## Competing interests

The authors declare that they have no competing interests.

## Authors’ contributions

HL and CDP designed the experiments. HL conducted the experiments. HL and CDP analyzed the data and prepared the manuscript. Both authors read and approved the final manuscript.
